# A novel nonparametric computational strategy for identifying differential methylation regions

**DOI:** 10.1186/s12859-022-04563-8

**Published:** 2022-01-10

**Authors:** Xifang Sun, Donglin Wang, Jiaqiang Zhu, Shiquan Sun

**Affiliations:** 1 Department of Mathematics, School of Science, Xi’an Shiyou University, X’an, 710065 Shaanxi People’s Republic of China; 2 Department of Biostatistics, School of Public Health, University of Michigan, Ann Arbor, MI 48109 USA; 3 Key Laboratory of Trace Elements and Endemic Diseases of National Health Commission, School of Public Health, Xi’an Jiaotong University, Xi’an, 710061 Shaanxi People’s Republic of China

## Abstract

**Background:**

DNA methylation has long been known as an epigenetic gene silencing mechanism. For a motivating example, the methylomes of cancer and non-cancer cells show a number of methylation differences, indicating that certain features characteristics of cancer cells may be related to methylation characteristics. Robust methods for detecting differentially methylated regions (DMRs) could help scientists narrow down genome regions and even find biologically important regions. Although some statistical methods were developed for detecting DMR, there is no default or strongest method. Fisher’s exact test is direct, but not suitable for data with multiple replications, while regression-based methods usually come with a large number of assumptions. More complicated methods have been proposed, but those methods are often difficult to interpret.

**Results:**

In this paper, we propose a three-step nonparametric kernel smoothing method that is both flexible and straightforward to implement and interpret. The proposed method relies on local quadratic fitting to find the set of equilibrium points (points at which the first derivative is 0) and the corresponding set of confidence windows. Potential regions are further refined using biological criteria, and finally selected based on a Bonferroni adjusted *t*-test cutoff. Using a comparison of three senescent and three proliferating cell lines to illustrate our method, we were able to identify a total of 1077 DMRs on chromosome 21.

**Conclusions:**

We proposed a completely nonparametric, statistically straightforward, and interpretable method for detecting differentially methylated regions. Compared with existing methods, the non-reliance on model assumptions and the straightforward nature of our method makes it one competitive alternative to the existing statistical methods for defining DMRs.

## Introduction

DNA methylation is a type of chemical modification of DNA nucleotides and is widely described as an epigenetic gene silencing mechanism. It has been discovered to affect changes in development, cell senescence, cancer, and other basic biological processes. Recently reported methods for whole-genome methylome have begun to clarify the importance of methylation location for gene transcription regulation [1, 2].

Cancer methylation studies could be one good motivating example. Altered methylation is a hallmark of the destabilization of genome integrity and function, and there are many aberrations in the methylomes of cancer cells while comparing with normal cells. Replicative senescence is a stable and tumor-suppressive state that has been shown to have similar methylomes to that of cancer cells, presumably because cancer cells inherit a number of the methylation landscape even after bypassing the senescence proliferation barrier. Therefore, identifying differentially methylated regions (DMRs) between normal proliferative cells and senescent cells could help elucidate epigenetic mechanisms that might promote malignancy, if the cells ultimately become cancerous by breaking through the proliferative barrier [3].

Bisulfite sequencing is the gold standard of whole-genome methylation sequencing, allowing for the measurement of methylation levels at the nucleotide resolution [4]. However, due to the complexities associated with whole-genome level data, the task of identifying differentially methylated regions remains one unresolved statistical problem. The main goal is to identify the possible statistically different methylation regions, taking into account the biological variation and controlling the false discovery rates. Such identified regions can later be compared against genome dictionaries to search for biological implications, or guide laboratory experiments.

Methods have been developed for different types of methylation data. Fisher’s exact test can be used if there are no biological replicates, but it is not insufficient for datasets with replications for its neglecting of taking common biological variation into consideration. Alternatively, regression-based methods make underlying assumptions about methylation levels and the associated variance, and subsequently perform statistical tests [5]. A popular package for performing pipeline analysis for defining differentially methylated regions is BSmooth. BSmooth applies a local-likelihood smoother to methylation data, and assumes that methylation counts follow a binomial distribution. A signal-to-noise statistic was developed, and tested for significance [5].

Although regression-based methods like BSmooth are popular, they are imperfect solutions. Predictions based on regression models are usually out of range. To remedy this problem, logistic regression or Poisson regression could be used, but the data will then often suffer from issues of over-or under-dispersion that will have to be corrected for statistically. The stringent conditions associated with regression-based analyses limit the confidence of results extracted from these analyses. Even more recent methods assume beta-binomial distributions, and some use Empirical Bayes methods to make further adjustments [6]. However, each of these methods carries with it a host of assumptions and is suitable for specific datasets.

Nonparametric model needs fewer or less stringent assumptions, and is more robust and not often seriously affected by extreme values, such as outliers. For example, Song, et al. reported a nonparametric method for identifying putative replication origins in yeast microarray data [7]. We adapted the idea and made various adjustments to enable it to be suitable for methylation data that comparing two different kinds of cells, with each had one or more replicates. In this paper, we propose one completely nonparametric, statistically straightforward, and powerful approach to identifying differentially methylated regions.

## Results

After smoothing and differencing, we obtained multiple differential DNA methylation curves on the testing data from chromosome 21. We excluded boundary sites, we analyzed methylation sites from 11,000 to 723,000. We identified 2402 equilibrium points and got their corresponding confidence windows according to the method described in Song, et al. [7]. After refining, 1085 candidate methylation regions were included. Finally, after conducting a *t*-test at a Bonferroni-corrected level of 4.6E−5, we identified 1077 differentially methylated regions.

**Fig. 1:**
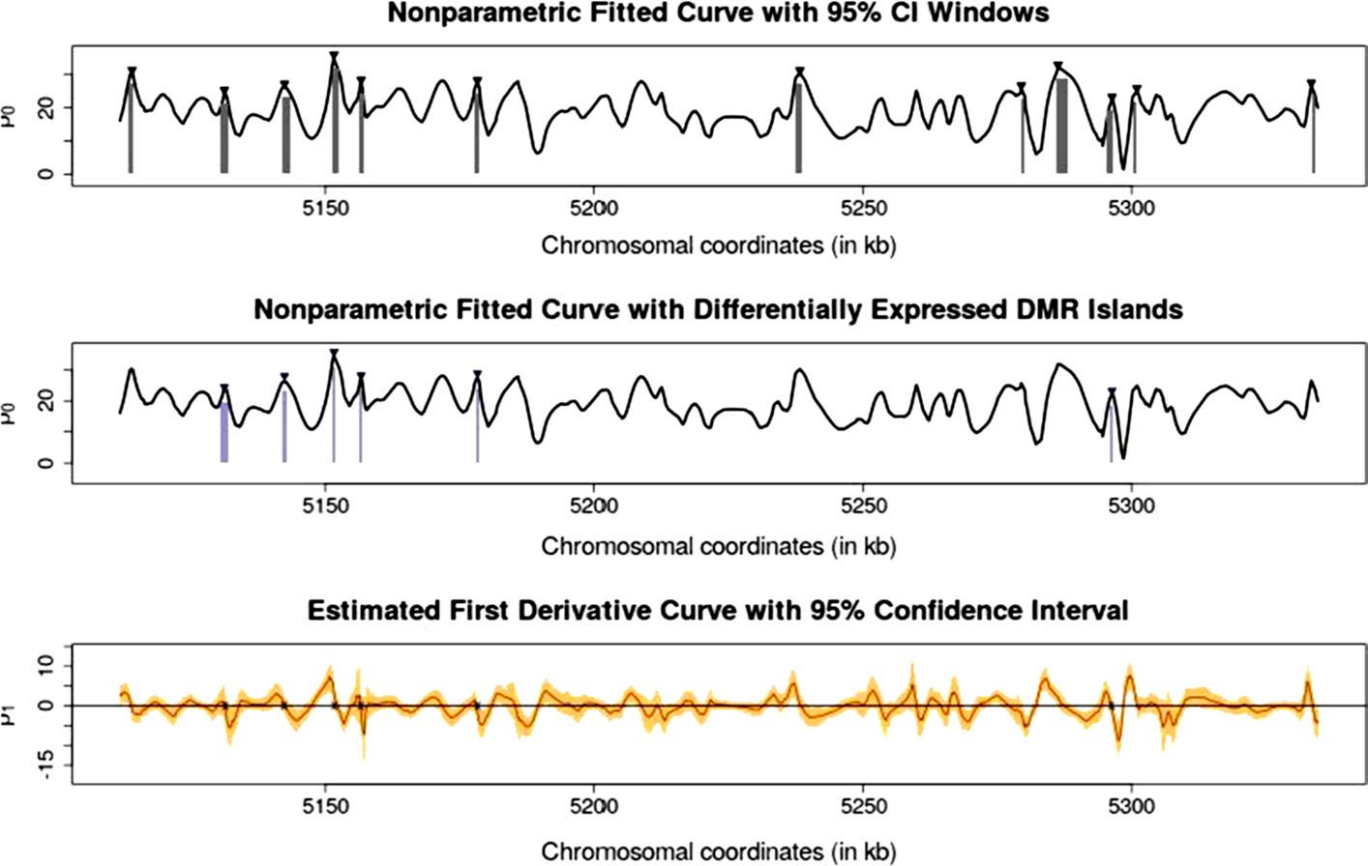
A sample segment of analysis on chromosome 21. Top panel: Potential equilibrium points and their confidence windows that were found through nonparametric local quadratic fitting (grey bars). Middle panel: Final differentially DNA methylated regions post-refinement and t-testing with Bonferroni correction (blue bars). Bottom panel: An estimated first derivative curve with 95% confidence interval (CI)

The top panel of Fig. 1 presents a representative segment of the difference curve of chromosome 21. 12 peaks were identified within this window, and 95% confidence windows were plotted. The middle panel in Fig. 1 shows the final list of differentially methylated regions post-refinement and *t*-test. with this window, approximately half of the peaks remained from the initial pool, and a majority of their confidence windows had been adjusted/narrowed. The bottom panel shows the corresponding predicted the first derivative plot of the difference curve. Equilibrium points that were selected by the local quadratic fitting method are represented by a closed circle. The stringent inclusion criteria were involved in defining a DMR. Although demanding, this requirement provides a robust level of power in identifying differentially methylated regions (DMRs).

## Discussion

In this paper, we identified a total of 1077 DMRs on chromosome 21 using proposed methods, which means that we could identify tens of thousands of DMRs on a whole-genome basis, basing on that the proportionality of MDRs is roughly estimated by the length of the chromosome. This is consistent with the findings of Cruickshanks, et al. [3] in Fig. 1, they found about 25,000 hypomethylated regions and 20,000 hypermethylated regions using the same dataset in the whole genome. In addition, the top panel of Fig. 1 shows an exclusively positive curve, confirming the previous observation that senescent cells, as compared to proliferative cells, are generally hypomethylated (stretches with negative difference curves exist, but were not shown).

As displayed in the top panel of Fig. 1, some local peaks were not chosen using this method. This is likely owing to our specific selection of smoothing bandwidth. Other choices of and smoothing kernels (i.e. tricube kernel, E-kernel) might identify a different set of peaks and have additional advantages (i.e. clean boundaries). A comparison between the top and middle panels of Fig. 1 illustrates that our refinement and testing method was successful in eliminating certain biologically infeasible regions, but it was not too stringent and still allowed the identification of a number of DMRs. Finally, although many equilibrium points were excluded in the process of refinement, the proportional difference post-refinement and post-testing was relatively small. The possible reason is that we used strict inclusion criteria to define DMR, that is, ten or more methylation sites are needed. However, this requirement brings a robust level of power in identifying differentially methylated regions.

One natural extension after generating the list would be to reference it with genome-wide data dictionaries with gene annotations, to see if any DMRs might have direct biologically meaningful indications. It could also be interesting to apply our method to other datasets in the literature and compare the performance with other reported analyses.

Our analysis was limited in some aspects, and we list the following areas for improvement. To begin with, methylation level is the ratio of methylated counts to total counts. Ratios of methylation over total counts such as 10/20 as compared to 1/2 lead to the same methylation level, even though the former arguably contains more information. This means that the main data indicators may be adjusted to enrich more original data information. In addition, the identification of DMRs is fundamentally a balance between controlling false discovery rates and not being overly stringent and ignoring true DMRs. If FDR is concerned, possible adjustments to decrease it could include requirements that all sites within the DMR meet minimum coverage (i.e. using total counts = 10), or requesting additional cutoffs (such as difference level > 25%) [8]. Finally, nonparametric methods are usually more expensive in terms of computation. We could reduce computational burden by running overlapping segments of chromosomes and processing segmental analyses downstream, but further work can be done to determine alternatives for faster computation. Finally, the proposed method is a nonparametric method that is generally more computationally expensive. In this paper, we were able to reduce the computation burden by running overlapping segments (i.e., divided into multiple chunks) of the chromosome and processing segmental analyses downstream, but further work can be done to determine computationally faster alternatives.

## Conclusions

In conclusion, despite certain limitations, we present in this paper a completely nonparametric, statistically straightforward and interpretable method for the detection of differentially methylated regions. Compared to existing methods, the lack of reliance on model assumptions and the straightforward nature of our method makes it a competitive alternative to existing statistical methods for defining DMRs.

## Methods of materials

### Dataset

One genome-wide methylation dataset from the National Center for Biotechnology Information (NCBI) Gene Expression Omnibus (GEO; Accession ID: GSM1181642) was used in our study [3], which contained the genome-wide methylation counts of proliferating and senescent cell types of the Coriell human fibroblast cell line (IMR90), with three replications of both two types. Sequencing data were obtained by whole-genome single-nucleotide bisulfite sequencing, and then aligned against a converted human reference genome (i.e., hg18), and the final methylated counts and total counts were determined by comparing with the reference genome.

Chromosome 21 was taken as the arbitrarily selected example in our analysis. A total of 724,212 methylation sites on this chromosome, after excluding 665 sites with a total methylation count of zero in all three replicates (leading to an uninterpretable methylation level), finally resulting in 723,547 total methylation sites for the downstream analysis.

### Statistical methodology

Our method begins by using the normalized methylation data, i.e., the ratio of the methylated and total counts to calculate a methylation level at each sequencing site. The procedure of the proposed method can be divided into three steps: smoothing of the difference, finding candidate regions, and refining and testing candidate regions.

### Smoothing of the difference

For each cell type (senescent vs proliferative), the site-specific weighted average methylation level (totaling all the methylated counts and dividing by the total counts at each site across replicates) was calculated. The difference in methylation level was obtained (proliferative minus senescent). The biological smoothness of the differential methylation levels is assumed, following examples in the literature [9, 10] Therefore, we use a Gaussian kernel to smooth the difference of weighted averages (the difference curve) between groups in a nonparametric way. We consider the hypothesis testing at a single coordinate to test whether a suspected site is a true equilibrium point, i.e.,

$$H_{0} :\dot{\theta }\left( {x_{0} } \right) = 0\; \, versus\; \, H_{1} :\dot{\theta }\left( {x_{0} } \right) \ne 0$$

where the rejection rule is that at this site $x_{0}$, we reject the null at 5% significance level.

Nonparametric smoothing depends on bandwidth selection. In our analysis, the standard deviation of the Gaussian kernel density is the same as its pre-specified bandwidth, *h*. For example, *h* = *0.5* kb means that a 2 kb window would cover a 95% region surrounding our smoothing location center. However, in order to ensure a minimum number of influence sites, especially in data poor regions, we expanded the bandwidth *h* until we obtain at least 70 influence points within the 95% kernel window. Therefore, *h* is a moving bandwidth to adapt to the sparsity of data at each chromosome location.

Due to the limitation of computational complexity, we divided the chromosome into segments of 2000 methylation sites (original segments) and ran the smoothing of the difference curves within these segments. In order to accurately predict the boundaries of these original segments, we analyzed the additional segments (which also had 2000 methylation sites) just centered exactly at the boundary between two original segments in the same way. The sizes and positions of the overlapping window were determined through sensitivity analyses, which analyzed the unbiasedness and accuracy of variance estimators. As a result, each additional segment overlapped 1000 methylation sites with the original segment on either side. The final predictions of the difference curve were a piecemeal combination alternating between predicted results of the original and additional segments, each contributing 1000 methylation site predictions from the center of its segment. The original segments from the boundary of the chromosome each contributed 1500 methylation site predictions on either side, but we discarded the analysis of the first and last 11,000 methylation sites to avoid inaccurate boundary estimations.

### Candidate regions

Consistent with the process of Song et al., the preliminary selection process of a candidate region consisted of searching for local extrema in the difference curve. In our analysis, a local extremum signified a locally elevated level of methylation difference between the two cell types. Local extrema were identified using local quadratic fitting. More specifically, nonparametrically estimated derivatives were used to identify the set of equilibrium points where $\theta^{{\left( {1} \right)}} = {0}$. A local quadratic fit (instead of a local linear fit) was used to reduce the edge effect. In the majority of cases, at local extrema, the sign of $\theta^{{\left( {1} \right)}} \left( x \right)$ will change from positive to negative. Because of the discreteness of our data, we will take the point whose first derivative is closest to zero and whose corresponding confidence interval contains zero. At a small percentage of points, we cannot directly observe the change of sign of $\theta^{\left( 1 \right)} \left( x \right)$. However, we can infer a local peak from confidence intervals containing zero.

To perform the local quadratic fitting, we first minimize the following objective function in the form of kernel weighted least squares at a given $x$ with respect to a quadratic function parameter vector $\alpha = (\alpha_{0} ,\alpha_{1} ,\alpha_{2} )^{T}$.

$$U(\alpha ) = \sum\limits_{i = 1}^{n} {\kappa_{h} (x_{i} - x)(y_{i} - \alpha_{0} - \alpha_{1} (} x_{i} - x))$$

where $\kappa_{h} (u) = h^{ - 1} \kappa (u/h)$ and $\kappa ( \cdot )$ is the standard normal kernel. At a given target coordinate $x,\hat{\theta }(x) = \hat{\alpha }_{0} ,\hat{\dot{\theta }}(x) = \hat{\alpha }_{1}$, we could estimate $\alpha = (\alpha_{0} ,\alpha_{1} ,\alpha_{2} )^{T}$ through:

$$\hat{\alpha } = (\hat{\alpha }_{0} ,\hat{\alpha }_{1} ,\hat{\alpha }_{2} )^{T} = \arg \min_{\alpha } U(\alpha )$$

If there are multiple confidence intervals in the sequence, the one whose first derivative is closest to zero will be chosen. Notably, for both of these cases, the selected site should have the largest* p*-value among the neighborhood potential equilibrium points.

Identifying local extrema by finding equilibrium points leads naturally to a way of constructing a confidence interval surrounding equilibrium points. Denote the confidence interval of the equilibrium point as $\left( {l,u} \right)$. For the majority of cases, we can invert the confidence intervals of the first derivative to find the corresponding positional bounds. That is, for each equilibrium point identified, we scan 10 methylation sites on either side. The upper confidence coordinate is the first location at which the first derivative is less than or equal to *l*, and the lower confidence coordinate as the first location at which the first derivative is greater than or equal to *u*. If we can only use this algorithm to find one position within our neighborhood, we appeal to the asymptotic symmetrical property, and define the other position by mirroring a number of sites on either side. We forego candidate regions where we cannot find either coordinate through this inversion method for further analysis (most of the cases are on the boundary).

### Refining and testing for DMRs

Many candidate regions will be selected in the second step (i.e., finding candidate regions), but not all of them are useful in practice. Therefore, the candidate regions were further refined through biologically motivated criteria. Candidate regions with very dispersed methylation sites (no neighboring methylation sites within 0.3 kb) were discarded. This was because it was recommended that each DMR should contain at least one CpG per 300bp^4^. Analogously, candidate regions with obvious clusters of methylation sites (where neighboring methylation sites were within 0.3 kb from each other) but were far between clusters were redefined as two or more candidate regions. Finally, candidate regions with less than 10 methylation sites were not analyzed further, adapting from methods that required at least 10 CpGs per candidate region [11].

All the candidate regions contained ten or more methylation sites, with all methylation sites less than 0.3 kb to their nearest neighbor. Each region was then tested for differential methylation status through a two-sided *t*-test, using the mean and standard deviation of the difference in methylation level across the methylation sites. Such a region was considered to be DMR if the *t*-test was significant at an *a*-level of $\frac{{0.05}}{{{{\# }}\;{\text{tested}}\;{\text{candidate}}\;{\text{regions}}}}$. The significance levels of the *t*-test was determined conservatively through a Bonferroni correction to reduce false discovery rates.

### Bandwidth selection

Bandwidth *h* is crucial for the smoothing algorithm as it determines the degree of smoothness. To determine the value of *h*, we adopted the minimum asymptotic mean integrated squared error method, which could choose the value of the bandwidth automatically by the observed data under a certain optimality criterion.

## Data Availability

The datasets supporting the conclusions of this article are included within the article and its additional files (GEO GSE48580; Accession ID: GSM1181642; https://www.ncbi.nlm.nih.gov/geo/query/acc.cgi?acc=GSM1181642). The source code is avaliable on GitHub (https://github.com/sqsun/NonparamDMR).

## Abbreviations

DMRs: Differentially methylated regions
FDR: False discovery rate
NCBI: National center for biotechnology information
GEO: Gene expression omnibus
CI: Confidence interval
